# Structural mass spectrometry techniques for characterisation of plant and algal proteins

**DOI:** 10.1111/tpj.70780

**Published:** 2026-03-14

**Authors:** Rhiannon Durant, Dušan Živković, Jani R. Bolla

**Affiliations:** ^1^ Department of Biology University of Oxford Oxford OX1 3RB UK

**Keywords:** cross‐linking mass spectrometry, hydrogen‐deuterium exchange mass spectrometry, native mass spectrometry, plant proteins, plant structural biology, structural mass spectrometry

## Abstract

Structural biology can offer valuable insights into the mechanisms and functions of key proteins within plant molecular and cellular systems. However, plant proteins present several specific challenges for structural analysis, including difficulties in expression and purification, significant intrinsic disorder, and extensive post‐translational modification. Structural mass spectrometry (MS) offers a complementary set of tools that can help overcome these obstacles and provide detailed structural and mechanistic information. In this review, we outline the principles and practical applications of the main structural mass spectrometry techniques, namely crosslinking, covalent labelling, hydrogen–deuterium exchange, and both intact (denaturing) and native MS. We also discuss recent case studies where structural MS has offered insight into the architecture, dynamics and interactions of proteins central to plant molecular and cell biology.

## Significance Statement

This review discusses the main structural mass spectrometry techniques and explains why they are especially effective for overcoming the specific challenges encountered in the structural biology of plant and algal proteins. It also showcases recent applications of structural mass spectrometry in plant and algal research and identifies areas where wider adoption of these methods could be particularly beneficial.

## INTRODUCTION

Structural biology uses structure–function relationships to understand the roles and mechanisms of macromolecules, especially proteins, in cells. Historically, most protein structures have been determined through X‐ray crystallography (XRC), cryo‐electron microscopy (cryo‐EM) and nuclear magnetic resonance (NMR) spectroscopy. XRC requires highly concentrated, pure, and homogeneous protein samples. It is further limited by the crystallisation bottleneck: finding conditions that promote crystal growth is an empirical and often slow process, and many proteins are difficult to crystallise altogether (Ochi et al., 2009). Cryo‐EM can tolerate greater heterogeneity than XRC and is much better suited to large complexes, but has a minimum size threshold and still preferentially captures relatively stable assemblies (Lander & Glaeser, 2021). Protein NMR spectroscopy can offer detailed information on dynamics in solution, but it requires stable isotope labelling, high amounts of pure protein and is limited to small proteins, as spectral assignment becomes intractable as protein size increases (Geue et al., 2022).

Although an increasing number of plant protein structures have become available in recent years, the structural biology of plant proteins still lags behind that of other organisms; only ~3% of structures from cellular organisms in the Protein Data Bank come from green plants (Berman et al., 2000). This is surprising given the importance of understanding plant systems for improving crop resilience to climate change and increasing food security. Several factors contribute to the relatively slow progress in plant structural biology. Gene editing in plants, including canonical model species, can be relatively difficult and slow (Mao et al., 2019). As a result, introduction of purification tags to extract proteins from native sources is often impractical, so plant proteins are usually expressed heterologously for structural studies. Common heterologous expression hosts such as *Escherichia coli* and yeast do not mimic the folding environment of the plant cell, and yields of correctly folded plant proteins are frequently poor. This makes it challenging to obtain the large quantities of pure material required for XRC in particular (Gorrec, 2021).

Plant proteins are also well known for their high levels of intrinsic disorder (Hsiao, 2022). This can prevent crystallisation or render regions invisible in cryo‐EM maps due to conformational flexibility. An additional difficulty, especially for complex assemblies isolated from endogenous sources, is that many key plant species still do not have fully annotated genomes because of polyploidy and other complexities (Sun et al., 2022). Therefore, methods that can determine protein identities and sequences directly from complex mixtures are essential, as some components may not yet be recognised from genomic data. Furthermore, plant cells are highly compartmentalised, and many compartments require transit peptides or signal sequences for protein targeting (e.g. the chloroplast stroma). Prediction tools for cleavage sites of these localisation signals exist, but are not always accurate, meaning that protein N‐termini in native complexes may differ from in silico predictions (Gould et al., 2024). At the same time, highly accurate protein structure prediction tools such as AlphaFold and RosettaFold have reduced the need to solve every structure de novo by XRC or cryo‐EM (Baek et al., 2021; Gould et al., 2024). What is often more useful now are methods that can verify predicted structures and assemblies, provide experimental restraints for integrative modelling, and report on conformational dynamics.

This is where the strengths of structural mass spectrometry (MS) become particularly relevant. Conventional MS for ‘omics’ applications, including metabolomics, lipidomics and proteomics, has been widely deployed in plant and algal research (Liu et al., 2024; Manickam et al., 2023; Yan et al., 2022). Traditional proteomics has been especially powerful for identifying proteins in complex samples and for comparative analyses between wild‐type and mutant organisms. We assume the reader is familiar with these approaches and direct interested readers to several reviews on plant proteomics (Balotf et al., 2022; Jiang et al., 2024; Mergner & Kuster, 2022).

Developments in instrumentation, chemistry and software over the past two decades have opened an additional avenue: the use of MS for protein structural characterisation. For example, modifications to mass analysers have extended the accessible m/z range, allowing detection of large protein complexes; increased resolution and mass accuracy are critical for distinguishing closely related proteoforms, such as those differing by PTMs (Ma, 2022; Pukala & Robinson, 2022). New additives and reagents have improved outcomes; short spacer‐length, enrichable and MS‐cleavable crosslinkers have enabled powerful crosslinking–MS workflows (Tang et al., 2021). Software advances, such as Bayesian deconvolution algorithms, have greatly improved the analysis of heterogeneous species that differ by PTMs or ligand binding and exhibit overlapping charge‐state distributions (Rolland & Prell, 2022).

Structural MS provides a diverse toolkit of techniques that generally tolerate low sample volumes, low protein concentrations and relatively modest purity, making them highly suitable for many plant and algal structural biology problems (Blackburn et al., 2022). These methods can provide distance restraints and solvent‐accessibility information that are directly useful for validating or refining computationally predicted structures (Biehn & Lindert, 2022; Orbán‐Németh et al., 2018). When combined with molecular dynamics simulations, structural MS data can be used to explore conformational dynamics and flexibility of protein structures (Dixon et al., 2022). This is particularly valuable for intrinsically disordered regions and for domains connected by flexible linkers, which are often invisible in crystallographic and cryo‐EM maps (Mitra, 2021).

Structural MS techniques can also confirm predicted oligomeric states and the architecture of complexes, or feed into integrative modelling pipelines that dock proteins together (Barth & Schmidt, 2020; Orbán‐Németh et al., 2018). They can probe interactions that are too weak, transient or heterogeneous to be captured in cryo‐EM structures. In parallel, MS can identify and characterise proteins within complex mixtures, including distinct proteoforms generated by backbone cleavage and post‐translational side‐chain modifications (PTMs) (Catherman et al., 2014; Habeck & Lermyte, 2023). Both covalent and non‐covalent adducts and ligands can be observed, even when their occupancy is low or their interactions are too weak to be detected by other structural methods (Barth & Schmidt, 2020).

**Figure 1:**
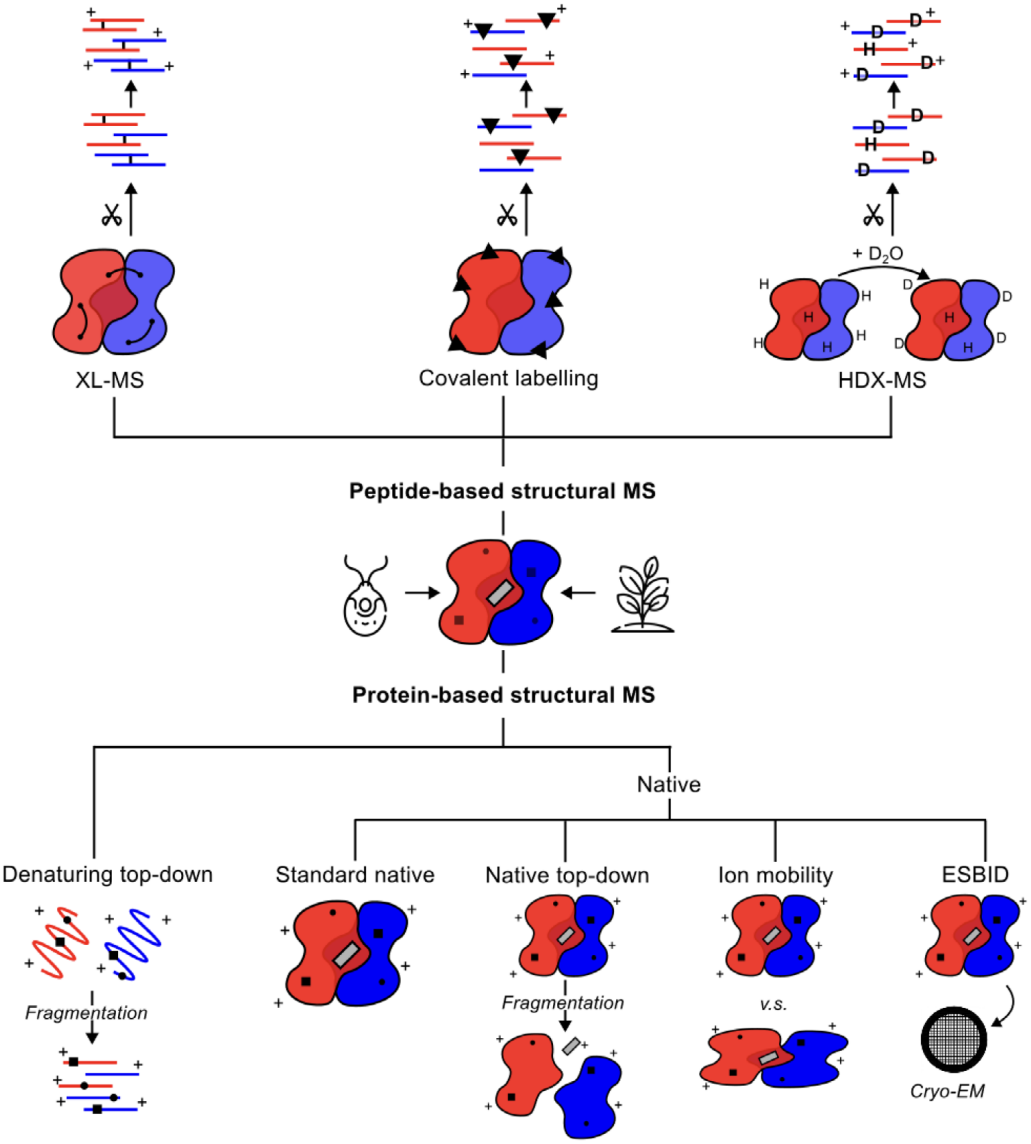
Key structural MS techniques. Structural MS techniques are generally classified into peptide‐based and protein‐based methods, depending on whether the protein is digested with proteases prior to MS analysis. Black squares and circles indicate covalent PTMs. The grey rectangle represents a non‐covalent ligand. D, deuterium; H, hydrogen. Scissors symbolise protease digestion.

These advantages position structural MS as an ideal set of methods for tackling many challenges in plant and algal structural biology. In the following, we introduce several key structural MS approaches, starting with peptide‐based methods: crosslinking MS (XL‐MS), covalent labelling and hydrogen–deuterium exchange MS (HDX‐MS), then progressing to protein‐based techniques: denaturing top‐down MS and native MS, including native top‐down, ion mobility, and soft‐landing workflows (Figure 1; Table 1). We briefly outline the principles and workflow for each method and highlight specific applications since 2020, where these approaches have been employed. Our focus is mainly on plant proteins, but we also include examples involving algae due to their evolutionary relatedness, especially for techniques in which no plant protein applications have yet been published.

**Table 1:** Advantages and uses of different structural MS techniques

Structural MS technique	Uses and strengths
XL‐MS	Provides distance restraints between residues, can be used to confirm structural predictions of individual complexes or multimers; can reveal changes in conformations or flexibility of domains; can characterise interactions of disordered regions
Covalent labelling	Identifies which regions/residues are solvent exposed; useful for finding protein–protein interaction interfaces or exploring changes in protein conformations
HDX	Reveals solvent accessibility and flexibility of peptide backbones plus rigidity of secondary structure; this can reveal conformational changes, disorder/order transitions, protein–protein binding interfaces, etc.
Denaturing top‐down	To differentiate between and identify proteoforms, which is very useful for studying combinations of PTMs
Traditional native MS	To explore oligomerisation states, protein–protein interactions, and ligand binding and their binding affinities.
Native top‐down	To determine identities of proteoforms within complexes, to reveal stable subcomplexes within higher order assemblies, and to identify bound ligands or even membrane protein‐bound lipids
Ion mobility	To explore conformational changes of folded proteins
Native ESIBD	To link mass information with spatial information; to enrich low‐abundance species and transient complexes for cryo‐EM analysis; to improve resolution of flexible surface regions via desolvation

## OVERVIEW OF THE MS ANALYSIS PROCEDURE

Mass spectrometers vary widely in configuration, and many instruments are custom‐built for particular applications. Nonetheless, most analyses follow a standard sequence. First, analytes are ionised, typically by electrospray ionisation (ESI or nano‐ESI) (Prabhu et al., 2023). Peptides or proteins are desolvated and charged as they are transferred from solution into the instrument's high vacuum under electric fields. Ions are often filtered by mass‐to‐charge ratio (m/z) in a quadrupole before entering the mass analyser. Several types of analysers are used in structural MS, including time‐of‐flight (TOF) analysers and Orbitraps, each with characteristic strengths in resolution, mass range, and speed (Boesl, 2017; Eliuk & Makarov, 2015). Many structural MS workflows include an additional fragmentation step in which selected ions are activated to dissociate into smaller fragments or to remove detergent micelles from membrane protein complexes. Fragmentation methods include collision‐induced dissociation (CID), higher‐energy collisional dissociation (HCD), surface‐induced dissociation (SID) and ultraviolet photodissociation (UVPD), among others (Macias et al., 2020). For a more detailed discussion of instrumentation and theory relevant to structural MS, we refer readers to several recent reviews (Eliuk & Makarov, 2015; Haag, 2016; Shuken, 2023; Sinha & Mann, 2020; Zhao et al., 2023).

## PEPTIDE‐BASED STRUCTURAL MS

The first class of techniques we consider operates at the peptide level: proteins are digested with proteases into shorter peptides before MS analysis. These methods (XL‐MS, covalent labelling MS and HDX‐MS) draw structural conclusions from patterns of crosslinks, side‐chain modifications or deuterium incorporation in peptides derived from the protein or complex of interest (Jiang et al., 2024).

### Crosslinking MS

Crosslinking MS uses bifunctional reagents that covalently link two residues within or between proteins. These crosslinkers act as molecular rulers: only residues within a distance compatible with the spacer length (i.e. the length of the added backbone bridging the two amino acid side chains (Graziadei & Rappsilber, 2022)) apart can be crosslinked. After crosslinking, proteins are digested and crosslinked peptides are identified by MS. The resulting inter‐ and intramolecular links provide distance restraints that report on the three‐dimensional proximity of regions within a protein (intra‐protein crosslinks) or on the relative arrangement of subunits within a complex (inter‐protein crosslinks) (Piersimoni et al., 2022). These restraints can be integrated with other data, for example, to guide docking simulations, to validate or refine de novo structural models, or to position low‐resolution or missing domains in cryo‐EM maps (Graziadei & Rappsilber, 2022).

Classical XL‐MS reagents contain two reactive groups that target specific side chains. Primary amines on lysine residues and N‐termini, or carboxyl groups of aspartate and glutamate, are commonly targeted. Many crosslinkers are now available with a range of useful features. Some are membrane‐permeable, enabling in situ crosslinking in intact cells prior to lysis and membrane solubilisation (e.g. DSS). Others incorporate an affinity handle to enable enrichment of crosslinked peptides, such as PhoX, which binds immobilised metal affinity chromatography (IMAC) columns (Steigenberger et al., 2019). A third class is MS‐cleavable, such as DSSO, which undergoes asymmetric cleavage in the collision cell to yield characteristic doublet fragment ions that greatly simplify identification of crosslinked species (Lee & O'Reilly, 2023). Crosslinkers also differ in spacer length. Shorter spacers provide tighter distance restraints but may capture fewer interactions; longer spacers increase the number of observable crosslinks but introduce more ambiguity to structural interpretation (Graziadei & Rappsilber, 2022).

After crosslinking, protein samples, ranging from highly complex cell lysates to purified proteins, are digested, most commonly with trypsin. Trypsin's well‐defined cleavage specificity facilitates the prediction of peptide masses (Saveliev et al., 2013). Because crosslinked peptides typically make up a small fraction of the digest (often <1%) and because high‐abundance unlinked peptides can suppress their ionisation, enrichment is almost always required. Size‐exclusion chromatography can be used, as crosslinked peptides generally have larger Stokes radii and elute earlier on average than unlinked peptides (Leitner et al., 2012). Strong cation‐exchange chromatography provides another route to enrichment: tryptic digestion leaves at least two positively charged residues in crosslinked peptides, which increases their retention on cation‐exchange media (Jiao et al., 2022). After enrichment, peptides are desalted, dried and resuspended before loading onto a reverse‐phase liquid chromatography (LC) column. As peptides elute from the column, they are ionised by ESI and analysed by MS/MS. Dedicated search engines and scoring algorithms are then used to identify crosslinked peptide pairs and derive the corresponding distance restraints.

#### Examples of XL‐MS in plant and algal structural biology

**Figure 2:**
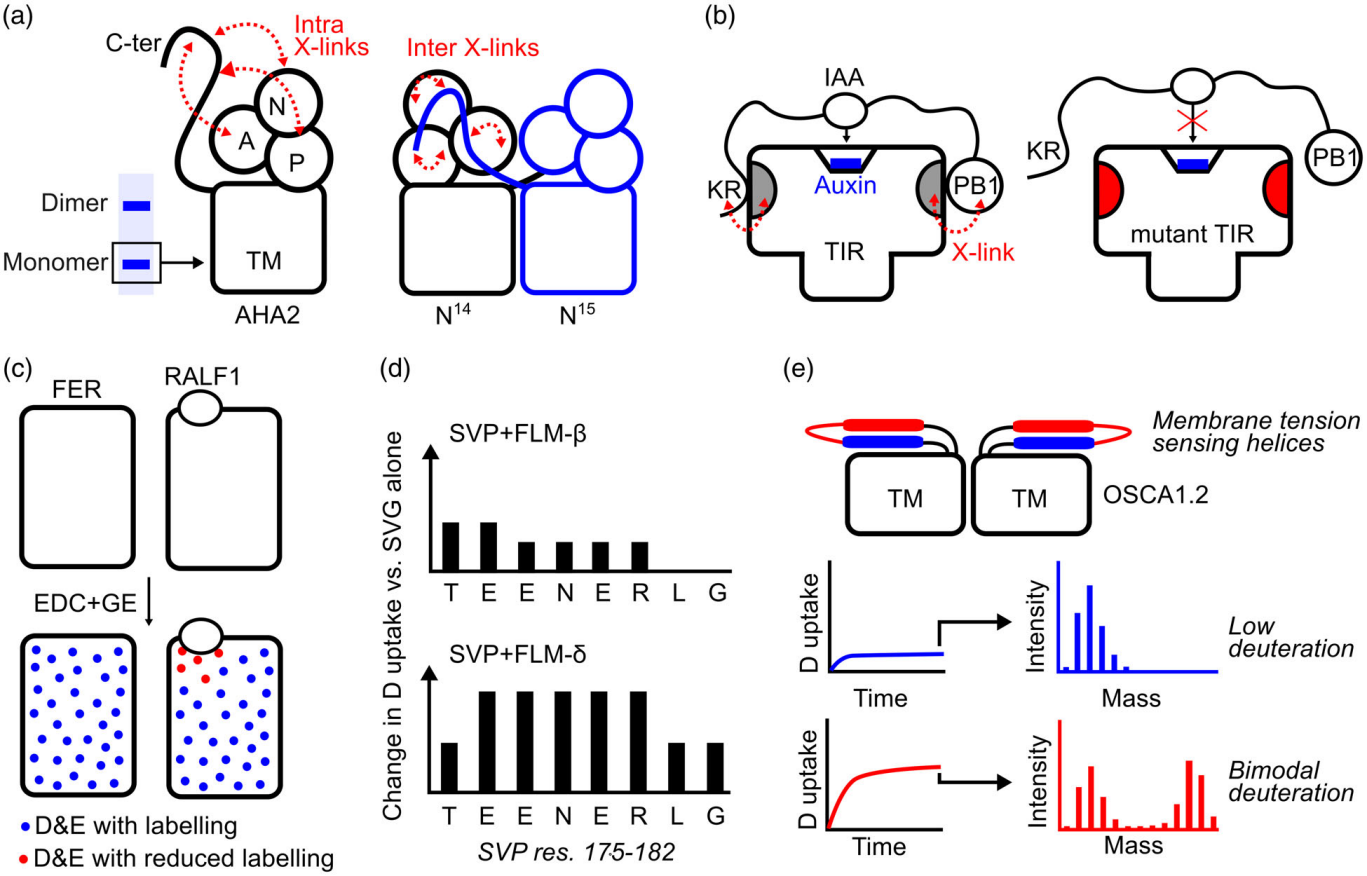
Examples of peptide‐based structural mass spectrometry techniques used on plant proteins. (a) To find intra‐strand crosslinks with the C‐terminal domain, monomer bands of AHA2 from SDS‐PAGE were cut out for analysis. A, actuator; N, nucleotide binding; TM, transmembrane; P, phosphorylation domains. To isolate inter‐strand crosslinks, N14 (black) and N15 (blue) labelled AHA2 were mixed and crosslinked; crosslinked peptides with mixed isotope types were detected and analysed. (b) Crosslinks were observed between a KR motif and the PB1 domain of IAA with clusters on either side of TIR (grey). Mutations in these clusters (red) decreased the responsiveness of TIR to auxin, likely due to reduced IAA binding. (c) Chemical footprinting with EDC and GE on FER with and without the presence of hormone RALF1 revealed five residues whose accessibility is reduced upon hormone binding. (d) Changes in deuterium (D) uptake of SVP peptides when combined with either FLM isoform are mostly similar; however, some regions show differences, such as residues 175–182, where FLM‐delta shows a greater increase in deuterium uptake and, therefore, solvent accessibility. (e) One OSCA1.2 membrane parallel helix (blue) shows little deuterium uptake in HDX, suggesting low solvent accessibility. Conversely, the adjacent loop and following helix (red) show rapid deuterium uptake. They also show a bimodal distribution of masses (representing the number of deuterium atoms exchanged) even after the full experiment time course, suggesting two distinct conformations with differing flexibility exist in solution.

XL‐MS has been used to probe the flexible C‐terminal regulatory domain of the Arabidopsis plasma membrane H^+^‐ATPase AHA2 (Nguyen et al., 2020). Crystal structures of AHA2 do not resolve this region, suggesting substantial disorder. Crosslinking with DSSO, combined with digestion with trypsin, GluC, and chymotrypsin, increased coverage and revealed that the C‐terminus forms crosslinks with the actuator subdomain and with other soluble domains of the protein (Figure 2a). To distinguish intra‐ from inter‐subunit contacts, the authors mixed ^14^N‐ and ^15^N‐labelled AHA2 prior to crosslinking and searched for crosslinked pairs where one peptide originated from each isotopic population. The presence of numerous “hybrid” crosslinks indicated that C‐terminal segments frequently interact with another protomer, supporting a model in which AHA2 forms autoinhibited dimers.

Elegant work by Albanese et al. (2020) used XL‐MS to characterise the interface between paired photosystem II–LHCII supercomplexes. Distance restraints derived from DSSO‐ and EDC‐mediated crosslinks were integrated with cryo‐EM structures of individual supercomplexes and predicted models of unresolved LhcB N‐termini in an integrative modelling framework. This revealed an intricate interface, anchored by the interaction between the LhcB4 N‐terminal loops from each supercomplex.

XL‐MS has also shed light on intrinsically disordered plant proteins. In one study, crosslinking with the MS‐cleavable reagent DSBU was used to probe the interaction between Aux/IAA and the auxin receptor TIR1 (Niemeyer et al., 2020). Aux/IAA is largely disordered, and its binding mechanism is unclear. Crosslinks were observed between the C‐terminal PB1 domain of Aux/IAA and a cluster of residues on one side of TIR1, and between a KR motif near the N‐terminus of Aux/IAA and a second cluster on the opposite side of TIR1 (Figure 2b). These constraints support a model in which Aux/IAA engages two spatially separated binding sites on TIR1, stretching the polypeptide so that the internal degron is properly positioned for ubiquitylation. Mutating residues within these TIR1 clusters largely abolished Aux/IAA‐dependent auxin responses *in planta*, validating the XL‐MS‐derived interface.

In Arabidopsis, a complementary large‐scale screen used the enrichable PhoX crosslinker to map PPIs (Trinh et al., 2025). IMAC enrichment increased the proportion of crosslinked peptides, and intra‐protein links were benchmarked against both PDB and AlphaFold models; >90% of links tested were within the expected maximal Cα–Cα distance, supporting the accuracy of the dataset. Many links revealed previously unknown PPIs. For example, the nucleoporin MOS7 was found to crosslink with histones, suggesting direct contacts between the nuclear envelope and chromatin that may contribute to genome organisation in plants.

### Covalent labelling MS

While XL‐MS imposes spatial restraints, covalent labelling reads out solvent accessibility and microenvironment, reporting conformational changes and interfaces under near‐native conditions (Liu et al., 2020). This technique (also called chemical foot‐printing) uses reagents that modify solvent‐exposed side chains. The pattern and kinetics of modification reflect the accessibility of different residues. Typically, time course experiments are performed in which the protein is exposed to the labelling reagent for increasing durations and modification at each site is quantified. Rapid and extensive labelling indicates high solvent exposure, whereas slow or incomplete labelling indicates burial within the protein core or at interaction interfaces (Limpikirati et al., 2018). Covalent labelling is usually applied comparatively: changes in solvent accessibility are measured between conditions, such as ±binding partner, ±ligand, or between mutants and close homologues. Loss of labelling upon complex formation can pinpoint residues at PPIs, whereas more subtle changes can report on conformational rearrangements propagated away from the binding site (Pan & Vachet, 2022).

There are two key classes of side chain modifiers that can be used for covalent labelling. The first are highly reactive reagents such as carbenes and hydroxyl radicals. Hydroxyl radicals can be generated directly from water solvent via X‐ray or γ‐ray sources, or by adding H_2_O_2_ and irradiating with a UV laser (Niu & Gross, 2019). They react with many side chains to yield a variety of oxidative modifications (Zhang et al., 2017). Similarly, carbenes generated from photoreactive precursors (e.g. photoleucine) can insert into C–H bonds and label a wide range of residues (Manzi et al., 2016). Carbenes are quenched by water on nanosecond timescales, which makes them suitable for probing very rapid folding dynamics (Konermann et al., 2010). The second class comprises residue‐specific reagents that target a single side‐chain type. Although these provide lower intrinsic spatial resolution, they simplify data analysis considerably. Examples include iodoacetamide for cysteine, EDC/glycineamide conjugation for carboxyl groups, diethyl pyrocarbonate (DEPC) for histidine and acetic anhydride for lysine (Mendoza & Vachet, 2009; Schmidt et al., 2017).

Following labelling, proteins are digested (commonly with trypsin) and the resulting peptides are separated by reverse‐phase LC. The modifications typically alter hydrophobicity and thus retention time, allowing separation of modified and unmodified peptides (Liu et al., 2020). Tandem MS is then used to localise modifications within peptides and thereby map solvent accessibility at near‐residue resolution.

#### Examples of covalent labelling MS in plant and algal structural biology

Covalent labelling has been used to compare the microenvironment of conserved lysines in photosystem II (PSII) complexes from cyanobacteria, red algae and spinach (Zhou et al., 2016). Reductive dimethylation of lysines using formaldehyde and NaBH_3_CN preserved positive charge, minimising structure perturbation. Among the five lysines conserved across all three species, four behaved similarly. PsbC‐K307 was the only site with markedly different labelling between species, with substantially higher modification in cyanobacterial PSII. This suggests subtle species‐specific differences in the local structure around this residue. Overall, the limited divergence at these conserved positions underscores the remarkable structural conservation of PSII across distant lineages.

Covalent labelling has also been used to define hormone–receptor interfaces. One study investigated the interaction between the plant peptide hormone RALF1 and its receptor‐like kinase FERONIA (FER) from Arabidopsis (Liu et al., 2018). EDC and glycine ethyl ester (GEE) were used to modify aspartate and glutamate residues in FER in the presence and absence of RALF1. Five FER residues showed marked decreases in labelling upon RALF1 addition, indicating direct occlusion or conformational rearrangements that reduced solvent exposure (Figure 2c). Because RALF1 lacks carboxylate side chains, the authors complemented this with XL‐MS using several crosslinkers, including DSSO. All three crosslinkers identified crosslinks between FER‐K60 and RALF1‐K114, and diagnostic DSSO fragments confirmed the identity of the crosslinked pair, together defining the orientation of RALF1 binding on FER.

### Hydrogen‐deuterium exchange MS

Covalent labelling emphasises side‐chain exposure; HDX‐MS instead probes the exchange of backbone amide hydrogens with deuterium. When proteins are diluted into buffer containing D_2_O, labile hydrogens exchange with deuterium, but on the timescale of typical HDX experiments, the relevant sites are backbone amide hydrogens (Vinciauskaite & Masson, 2023). Exchange rates depend on solvent accessibility and hydrogen‐bonding status and are thus sensitive to secondary structure and dynamics. Incorporation of deuterium leads to a mass increase that can be measured by MS. Importantly, replacing hydrogen with deuterium minimally perturbs protein structure and dynamics, in contrast to the larger modifications introduced by many covalent labelling reagents (Konermann & Scrosati, 2024). As with covalent labelling, HDX‐MS is usually used in a comparative mode, monitoring changes in deuterium uptake upon ligand binding, complex formation, mutations or changes in solution conditions. Regions that become protected upon binding often correspond to interfaces, while changes in exchange in the distal areas can reveal allosteric communication or disorder–order transitions (Parson et al., 2022; Woods et al., 2024).

HDX is typically automated using a robot coupled to the mass spectrometer (Masson et al., 2019). Protein samples are rapidly diluted into D_2_O buffer, and aliquots are withdrawn at defined time points spanning seconds to hours. Exchange is quenched by lowering the pH to ~2.5–3.0 and cooling to ~0°C, conditions that dramatically slow back‐exchange (Peterle et al., 2022). Subsequent digestion and LC steps are carried out under these “cold acidic” conditions and as rapidly as possible to minimise deuterium loss.

Pepsin immobilised on a column is commonly used for digestion, as it retains activity at low pH. Peptides are separated on a short reverse‐phase LC gradient before entering the mass spectrometer. The resulting spectra are complex, with overlapping isotopic envelopes for many peptides, and specialised software is used to track centroid shifts over time and calculate deuterium uptake (Rand et al., 2014). Initial identification of peptides generated by peptic digest is usually performed on non‐deuterated samples using standard LC–MS/MS with CID; fragmentation of deuterated peptides is generally avoided, as it can induce deuterium scrambling (Rand et al., 2014).

#### Examples of HDX in plant and algae structural biology

HDX‐MS has been applied to dissect activation mechanisms of plant cysteine dioxygenases (PCOs), which regulate hypoxia responses by oxidising the N‐termini of ERF‐VII transcription factors under normoxic conditions. Peptidomimetics based on the N‐terminal sequence of ERF‐VII Rap2.12 were designed to compete for binding and were expected to inhibit AtPCO4. Surprisingly, these peptidomimetics increased AtPCO4 activity. HDX‐MS was used to compare deuterium uptake in PCO4 with and without peptidomimetic (Latter et al., 2025). As anticipated, exchange decreased at the substrate‐binding site, indicating protection upon binding. However, increased exchange was observed in two remote regions, including a putative O_2_ tunnel leading to the active site. These changes support a model in which substrate binding promotes conformational changes that enhance O_2_ access, thereby increasing catalytic activity.

HDX‐MS has also been used to probe how alternative splicing modulates transcription factor behaviour. One study investigated SVP, a flowering time regulator, and its interaction with two splice variants of the MADS‐box protein FLM (Jin et al., 2022). FLM‐β promotes SVP nuclear localisation and repression of flowering, whereas FLM‐δ binding correlates with cytosolic localisation and enhanced ubiquitylation of SVP. HDX‐MS revealed that both FLM variants induce conformational changes in SVP, but the changes were more pronounced with FLM‐δ (Figure 2d). The data support a scenario in which FLM‐δ binding exposes degrons that target SVP for proteasomal degradation.

In work on mechanosensitive ion channels, HDX‐MS was used alongside cryo‐EM and molecular dynamics to study the rice OSCA1.2 channel (Maity et al., 2019). Cryo‐EM provided a high‐resolution structure of the dimeric complex, and simulations suggested that pairs of helices running parallel to the membrane transmit lateral membrane tension to the pore‐lining helices. HDX‐MS showed very low deuterium uptake for one of these helices, consistent with conformational rigidity required for signal transmission. In contrast, the partner helix and intervening loop displayed a bimodal uptake pattern over the full‐time course, indicating the presence of two distinct conformational states at rest (Figure 2e).

Finally, HDX‐MS has highlighted functional divergence between plant and animal homologues. In mammals, the C‐terminus of Rab escort protein (REP) binds unprenylated Rab proteins and is essential for their modification with the geranyl group (Gutkowska et al., 2021). HDX‐MS showed no significant change in deuterium uptake in the C‐terminal region of plant REP upon Rab binding, suggesting that this segment does not contribute to interaction. Deletion of the REP C‐terminus in vivo had minimal phenotypic effect, in contrast to a full REP knockout, confirming that plant REP uses a distinct binding mode.

## PROTEIN‐BASED MS

In contrast to peptide‐level methods, protein‐based structural MS analyses intact proteins (and complexes) without prior digestion. This requires instruments capable of transmitting and detecting high m/z ions (Prabhu et al., 2023). Two main approaches are particularly relevant here: denaturing top‐down MS, in which intact proteins are analysed under denaturing conditions, and native MS, in which intact proteins and complexes are maintained in, or close to, their native conformational states.

### Denaturing top‐down MS

Under denaturing conditions, top‐down MS (TD‐MS) enables intact proteoforms, specific molecular forms of proteins defined by sequence and PTMs, to be “weighed” and distinguished (Schaffer et al., 2019). Individual proteoform ions can be isolated and fragmented to provide sequence information and localise PTMs. This is especially powerful when combinations of modifications are functionally important. In peptide‐based workflows, digestion obscures which peptides originate from which proteoform, so combinatorial patterns of PTMs are often lost (Borotto, 2024). In addition, N‐ or C‐terminal truncations are difficult to identify unambiguously by bottom‐up MS because missing peptides could reflect poor ionisation or incomplete digestion rather than true proteolysis (Klein et al., 2018). Analysis of intact proteins can overcome these challenges.

For complex mixtures, LC is often used prior to MS to separate different proteins and proteoforms. Unfolded intact proteins usually have greater hydrophobicity than short peptides. Therefore, shorter chain lengths are used for reverse‐phase LC columns used to separate out samples for protein‐based versus peptide‐based MS, for example C4 columns rather than C18 columns (Finoulst et al., 2011). Upon ESI, intact protein ions are transmitted into the mass analyser, and selected charge states corresponding to a given proteoform are isolated. These ions are then fragmented, typically by CID, HCD, electron capture dissociation (ECD) or electron transfer dissociation (ETD), to generate a series of b/y‐type (CID/HCD) or c/z‐type (ECD/ETD) fragments (Brodbelt, 2022). Matching these fragments to theoretical patterns enables the assignment of sequences and PTMs.

#### Examples of denaturing TD‐MS in plant and algae structural biology

*Chlamydomonas reinhardtii* is known for its ability to silence foreign DNA, complicating its use as a photosynthetic host in biotechnology (Neupert et al., 2020). TD‐MS of histones has been used to investigate how combinatorial PTM states contribute to chromatin compaction and gene silencing (Rommelfanger et al., 2021). Intact histone masses defined distinct proteoforms, including 24 different H3 proteoforms. Fragmentation revealed two main H3 populations: a majority species carrying H3K4me1 with little or no additional N‐terminal acetylation, and a minority bearing H3K4me3 with extensive acetylation. H3K4me1 had previously been associated with repressive chromatin, and the predominance of this proteoform suggested that much of the Chlamydomonas genome resides in a repressive state. The data further suggested that H3K4me1 may preclude broader N‐terminal acetylation in this organism (van Dijk et al., 2005).

Denaturing TD‐MS has also been used for an unbiased broad screen for new PTMs and proteoforms in Arabidopsis (Wang et al., 2023). Proteins from leaf tissue and isolated chloroplasts were fractionated by size‐exclusion chromatography and analysed by TD‐MS. Mass shifts relative to predicted unmodified proteins were assigned to different PTMs, revealing distinct patterns across subcellular compartments; for example, chloroplast proteins were more frequently oxidised than those in total leaf extracts, highlighting potential redox sensors. The approach also allowed empirical determination of mature N termini. Among 343 proteins with detectable N‐terminal truncations, only 253 matched the predicted cleavages from TargetP 2.0, underscoring the limitations of current targeting algorithms. Rubredoxin A, for instance, was predicted to have its chloroplast transit peptide cleaved at residue 59, but the vast majority of observed proteoforms began at residue 55.

**Figure 3:**
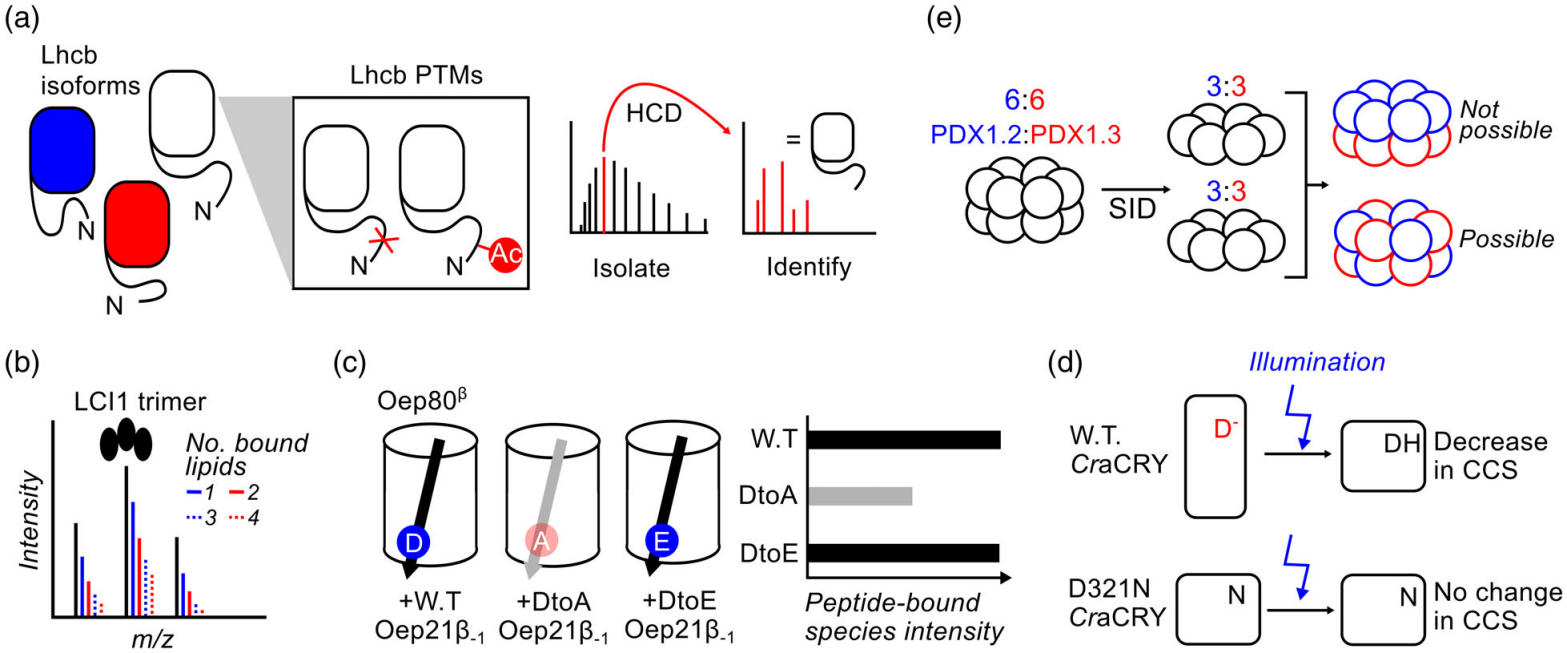
Examples of protein‐based structural MS technique use on plant and algal proteins. (a) Purified PSII‐LHCII contained heterogeneous mixtures of Lhcb proteoforms. After separation by mass, isoforms were assigned based on sequence information obtained through HCD fragmentation. Further analysis of fragment masses enabled identification and localisation of PTMs. (b) Black peaks represent the charge‐state series of the unbound LCI1 trimer. Coloured peaks adjacent to the main charge series represent mass increases corresponding to sequential binding of up to 4 lipids. (c) Oep80^β^ was combined with wild type (W.T.) and mutant peptides (arrows) from Oep21a's C‐terminal β strand (β_−1_) and analysed by native MS. An aspartate to alanine mutant (grey) showed weaker interaction. (d) Wild type (W.T.) CraCRY exhibited reduced CCS and thus increased compaction upon activation by illumination. Mutation of an aspartate to arginine to mimic protonation (D321N) resulted in low CCS in both the dark and illuminated states, suggesting that illumination‐induced protonation triggers conformational changes that activate CraCRY. (e) Mixed dodecamers of PDX1.3 (red) and PDX1.2 (blue) were weighed by native MS, then fragmented horizontally by SID. The masses of the resulting hexamers indicated subunit ratios consistent with the dodecamer (e.g. 6:6 and 3:3 in this example), demonstrating even distribution of subunits across both hexamers.

In the aforementioned PSII‐LHCII paper by Albanese et al. (2020), denaturing TD‐MS was used to characterise the heterogeneity of LHCII subunits within isolated paired PSII‐LHCII supercomplexes. Because the N‐terminal loops of these subunits, proposed to mediate inter‐supercomplex contacts, are heavily post‐translationally modified, protein‐level information was essential. TD‐MS workflows isolated individual LhcB proteoforms, identified the corresponding isoforms by fragmentation and mapped N‐terminal acetylations and truncations (Figure 3a). This information was incorporated into integrative models of the paired supercomplexes, constraining the conformation and interactions of the N‐terminal loops.

### Native MS (and its derivatives, including ion mobility, native top‐down and soft landing)

Native MS is unique among the structural MS techniques in that it preserves near‐native protein folds and non‐covalent assemblies in the gas phase (Tamara et al., 2022). Under suitable conditions, oligomeric states, PPIs, protein–ligand and protein–ion interactions, and specific lipid binding can all be maintained and directly observed (Bennett et al., 2022; Kumar et al., 2024). Native MS is label‐free, does not require immobilisation or extensive modifications, and can detect even weak or transient interactions (Boeri Erba & Petosa, 2015; Cubrilovic et al., 2012). Importantly, it tolerates a degree of heterogeneity and can quantify co‐existing stoichiometries or ligand occupancies that may be averaged out in methods such as XRC or cryo‐EM (Bennett et al., 2022).

The distributions of charge‐state series for a protein or complex in native MS analysis can also provide information on its folded state. Highly charged, broad distributions generally indicate unfolded or extended species with many accessible protonation sites, whereas compact, folded proteins typically yield relatively narrow, near‐Gaussian charge distributions (Lawrence et al., 2025). Ligand binding shifts the m/z of these charge‐state series. Under substoichiometric binding, clusters of peaks appear at each charge state, corresponding to apo and increasingly liganded species (Barth & Schmidt, 2020).

Protein folding and compactness can also be used to separate ions in native MS, via ion‐mobility (IM) MS. In drift‐tube IM, ions traverse a gas‐filled cell under a weak electric field that opposes the gas flow. Their drift times are used to calculate collisional cross sections (CCS), with more compact ions exhibiting shorter drift times and lower CCS values (Christofi & Barran, 2023). IM can thus distinguish conformers or complexes that share the same m/z but differ in overall shape, providing an additional structural dimension. IM is used in both denaturing and native workflows, but is particularly informative in native MS, where conformational changes often translate into CCS shifts.

Top‐down MS, mentioned above, can also be extended to intact complexes in their native state (nTD‐MS). Here, intact protein assemblies are ionised under native conditions, and individual subunits or proteoforms are ejected and subsequently fragmented. This can proceed in two main ‘flavours’. In one, the focus is on measuring the intact masses of subunits released from the complex, which informs on composition, stoichiometry and stability. In the other, the ejected subunits are isolated and fragmented to obtain MS/MS spectra that define the sequence and PTMs of each proteoform (Smith & Kelleher, 2013). Although originally limited by m/z range and fragmentation efficiency, improvements in instrumentation and methods such as UVPD and SID have enabled nTD‐MS of increasingly large assemblies (Greisch et al., 2019; Stiving et al., 2019). nTD‐MS is particularly valuable for dissecting compositional heterogeneity in complexes and for identifying bound ligands or cofactors (Quinn et al., 2023). SID represents a particularly informative variant of nTD‐MS. Unlike CID, which tends to eject highly charged monomers and leave a residual complex, SID promotes more symmetrical breakage and can yield intact subcomplexes that better reflect the native assembly pathway. Although most current nTD‐MS configurations still struggle to provide deep MS^3^‐level sequence coverage of all subunits in very large complexes, combining nTD‐MS with denaturing TD‐MS and bottom‐up proteomics can give a remarkably complete picture of proteoform composition and connectivity.

Finally, soft‐landing electrospray ion beam deposition (ESIBD) allows mass‐selected ions to be gently deposited onto surfaces under vacuum (Rauschenbach et al., 2016). In the context of structural biology, native ESIBD can be used to transfer intact protein complexes from solution into the gas phase by native ESI, mass‐select specific oligomeric states or ligand‐bound forms, and then “land” these species onto cryo‐EM grids for imaging. Recent work has demonstrated the feasibility of combining native MS with ESIBD and cryo‐EM to obtain high‐resolution structures of soft‐landed complexes, directly linking the chemical specificity of MS with the spatial resolution of cryo‐EM (Esser et al., 2024). In principle, this approach allows enrichment of low‐abundance species, such as rare oligomeric states or specific proteoforms, before imaging, overcoming one of the limitations of conventional grid preparation in which species are present in proportion to their equilibrium abundances in solution (Fremdling et al., 2022).

Prior to native MS analysis, protein samples must be exchanged into MS‐compatible buffers. Many salts and additives that stabilise proteins in solution are non‐volatile and must be removed to obtain resolvable spectra. For denaturing MS, proteins are simply exchanged into volatile organic solvents before analysis, but these conditions are incompatible with native structures. Native MS therefore uses aqueous solutions of volatile salts (most commonly ammonium acetate) to stabilise proteins and complexes (Li et al., 2025). Some proteins remain unstable under such conditions, necessitating empirical optimisation of pH, ionic strength, and additive composition. Buffer exchange is usually performed offline by spin columns or dialysis.

Sample preparation of membrane proteins for native MS requires particular care, as many detergents are poorly compatible with MS. Detergents, such as tetraethylene glycol monooctyl ether (C8E4), G1‐OGD and lauryldimethylamine‐N‐oxide (LDAO), which can be efficiently removed in the gas phase, are often favoured (Lawrence et al., 2025). Native MS has also been successfully performed from membrane mimetics such as peptidiscs or even directly from native membranes, enabling characterisation of endogenous complexes and bound lipids (Deedwania et al., 2026; Oluwole et al., 2023).

After buffer exchange, samples are introduced into the mass spectrometer, generally via nano‐ESI using gold‐coated glass capillaries (Leney & Heck, 2017). This generates ions with substantial kinetic energy, especially for large complexes. Without collisional cooling, these ions can be lost before analysis. Native MS instruments, therefore, operate with higher gas pressures in the front end to promote collisional cooling and energy dissipation (Chernushevich & Thomson, 2004). In native MS analysis, promoting lower charge states of proteins and complexes improves their stability and helps preserve their native states. However, this results in high mass/charge values, especially for large complexes. Native MS spectrometers are modified to include quadrupoles that operate at lower frequencies, enabling even high m/z ions to maintain stable trajectories through them and enter the downstream mass analysers (Tamara et al., 2022).

Additional steps can be taken to obtain useful native MS spectra from more challenging samples. For example, addition of charge‐reducing chemicals, such as TMAO, can also artificially shift proteins to lower charge states (Patrick & Laganowsky, 2019). This can reduce repulsion and unfolding as well as improve resolution, as the absolute m/z value difference between adjacent charge state peaks will be greater (Rolland & Prell, 2022). In addition, heterogeneity arising from PTMs and adventitious adducts can broaden and overlap charge states, complicating deconvolution. A more recently developed technique, charge‐detection MS, partially overcomes this by measuring both the m/z and charge of individual ions, enabling direct calculation of their masses and providing mass distributions for highly heterogeneous samples (Jarrold, 2022).

#### Examples of native MS (and its derivatives) in plant and algae structural biology

Native MS (and derivatives) have begun to be applied to specific plant and algal systems, often in combination with other structural methods.

After the determination of the crystal structure of the trimeric algal inorganic carbon transporter LCI1, Kono et al. (2020) used native MS to confirm that the protein remains trimeric in solution. The dominant charge‐state series corresponded to a trimeric species, and additional peaks revealed binding of up to four lipids per trimer (Figure 3b). In a similar vein, van den Berg et al. (2021) analysed a truncated form of the *Oryza sativa* silicic acid transporter NIP2 that had been crystallised successfully. Native MS showed that the truncated protein assembles as a tetramer, as does the full‐length protein, supporting the physiological relevance of the crystallographic construct.

Native MS can also interrogate sequence specificity in PPIs. β‐barrel assembly proteins BamA and Sam50 recognise substrates via C‐terminal β‐signal sequences, and the chloroplast outer envelope protein Oep80 is predicted to use an analogous mechanism. Native MS of refolded Oep80, combined with candidate β‐signal peptides from putative substrates, revealed strong binding to two peptides. Mutating a conserved penultimate acidic residue in these β‐signals to alanine markedly reduced the intensity of peptide‐bound Oep80 peaks, implicating this residue as a key determinant of β‐signal recognition (Figure 3c) (Durant et al., 2025).

IM‐MS has been used to probe the conformational changes of the *Chlamydomonas* cryptochrome *Cr*aCRY upon light excitation of its chromophore (Zangl et al., 2024). CraCRY ions were injected into an IM–MS instrument with or without prior blue‐light illumination. Light exposure decreased the CCS, indicating a more compact conformation. A D321N mutant, predicted to mimic constitutive protonation at a key active‐site residue, showed no CCS change upon illumination, consistent with this variant being locked in a ‘lit‐state‐like’ conformation (Figure 3d).

nTDMS has been used to characterise the diversity of the phycobilisome (light‐harvesting) complexes, with a multifaceted approach to characterise the gamma chains, which are buried deep inside the complexes and thus difficult to isolate for fragmentation. Besides this, the authors managed to discern between the isobaric, covalently bound chromophores bound to the phycobilisome complex subunits and determine their exact sites (Tamara et al., 2019). Furthermore, it was shown that it is possible to study the phycobilisome complex using UVPD for dissociation (Greisch et al., 2019).

In the cryo‐EM structure of a dodecameric complex of *A. thaliana* enzyme PDX1.3 with pseudoenzyme PDX1, the two different isoforms are indistinguishable, suggesting the solution ensemble is a mixture of different permutations of these subunits. Samples were sprayed natively, and ratios of PDX1.3 to PDX1.2 were determined based on total complex mass. Then, the dodecamer was fragmented with SID. The resulting subcomplexes were mostly hexamers, indicating horizontal cleavage was occurring. The hexamer masses were usually half that of the precursor dodecamer, indicating even distribution of isoforms across the two hexameric rings in the dodecamer (Figure 3e) (Novikova et al., 2021). This exemplifies how nTD‐MS can reveal information about complex subunits that cannot be obtained from crystal or cryo‐EM structures.

Native MS combined with ESIBD and cryo‐EM has been applied to plant RuBisCO, demonstrating that native ESIBD can deliver intact complexes suitable for high‐resolution structure determination (Eriksson et al., 2025). In the reported work, an *A. thaliana* RuBisCO complex prepared by native ESIBD yielded a cryo‐EM structure at ~2.6 Å resolution, comparable to structures obtained by conventional sample preparation. Interestingly, local resolution at protein surfaces was improved, likely because gas‐phase dehydration promotes intramolecular interactions in labile loops that would otherwise be stabilised by solvent. This underscores the potential of MS‐guided cryo‐EM workflows to link detailed chemical information, such as proteoform composition or ligand occupancy, with high‐resolution structures of specific assemblies.

## CONCLUSION

Structural MS provides a diverse toolkit of techniques for addressing outstanding questions in plant molecular biology. Applications range from identifying protein binding partners and mapping PPIs, through determining complex stoichiometry and proteoform composition, to elucidating ligand‐induced conformational changes and dynamics. These techniques complement, and in some cases can substitute for, XRC, NMR, and cryo‐EM, for example, by validating models generated by structure prediction algorithms such as AlphaFold, or by providing distance and accessibility restraints for integrative molecular docking and dynamics simulations.

Structural MS approaches are particularly well‐suited to plant proteins. They tolerate sample heterogeneity and lower purity, which is advantageous where obtaining high quantities of pure protein is challenging. They are uniquely suited to probing intrinsically disordered proteins and regions, which are widespread in plant systems yet largely invisible to traditional structural methods. Protein‐level approaches can simultaneously yield structural and sequence information, including proteoform‐specific PTM patterns and N‐ or C‐terminal processing, which is especially valuable in species with complex, incompletely annotated genomes.

Despite the examples discussed here, structural MS remains underutilised in plant and algal research. There is significant potential for expansion. Plant molecular biologists are encouraged to consider structural MS when addressing complex structural questions, whether through collaboration with MS specialists or by developing internal capabilities. Likewise, structural MS experts should recognise that their tools and expertise could greatly impact plant biology, where many structural questions remain, hindering our efforts to engineer crops for improved yield under changing climate conditions. We anticipate that structural MS will become an increasingly important part of plant and algal biology in the coming years, helping to resolve longstanding questions about protein architecture, dynamics, and interactions, and enabling insights that are difficult or impossible to achieve with other techniques alone (Boxes 1 and 2).

Box 1 **Summary bullet points**

- Progress in the structural characterisation of plant and algal proteins has lagged behind that of many other systems.
- Contributing factors include difficulties in heterologous expression and purification, high levels of intrinsic disorder, extensive post‐translational modifications and incompletely annotated genomes.
- Structural mass spectrometry (MS) provides advantages over other structural techniques that can help overcome some of these challenges, such as tolerating sample heterogeneity, detecting transient and weak interactions, and linking structural information directly to sequence and proteoform‐level analysis.
- Key structural mass spectrometry techniques include crosslinking MS, covalent labelling, HDX, denaturing top‐down MS, and native MS.
- Structural MS has already been used on a range of plant and algal proteins, but the field remains relatively underexplored, and there is considerable potential for broader and more systematic application of these technologies.

Box 2 **Open questions**

- Which structural problems in plant biology are currently limited by access to sufficient quantities of pure protein, and where could structural mass spectrometry provide a practical alternative?
- Where is information on the dynamics and interaction networks of intrinsically disordered or low‐complexity plant protein regions most urgently required, and to what extent can bottom‐up structural mass spectrometry address these questions?
- Which questions in plant signalling, metabolism, or stress responses require detailed analysis of heterogeneous proteoforms, and where could denaturing top‐down mass spectrometry be particularly useful?
- What unresolved questions in plant biology could be investigated further through quantitative analysis of non‐covalent complexes and assemblies using native mass spectrometry and related gas‐phase techniques?
- What practical, technical, or conceptual barriers currently hinder the adoption of structural mass spectrometry by plant and algal scientists, and how might these be mitigated through method development, training or infrastructure?

## CONFLICT OF INTEREST

The authors have no competing interests.

## Data Availability

Data sharing is not applicable to this article as no datasets were generated or analysed during the current study.
